# Editor's Note: 2012 Reviewers of the Year

**DOI:** 10.1289/ehp.121-a41

**Published:** 2013-02-01

EHP received nearly 1,500 papers during 2012, and nearly 500 of those papers were sent out for peer review. Each paper is evaluated by at least 2 reviewers for scientific quality and content. EHP is very grateful to the more than 1,000 reviewers who were part of the peer-review process. A list of those reviewers is available on the journal’s website (http://ehp.niehs.nih.gov/list-of-reviewers-2012/). EHP would also like to recognize our top 12 Reviewers of the Year. These individuals completed at least 7 papers during the year and received high ratings from the Associate Editors for their timeliness and the quality of their reviews. The EHP 2012 Reviewers of the Year are Sara Adar, Andrea Baccarelli, Aaron Barchowsky (not pictured), Joe Braun, David Carpenter, Ulrike Gehring, Gerard Hoek, Barbara Hoffmann, Matthew Longnecker, Roger McClellan, Allan Smith, and Leonardo Trasande. EHP is grateful to these and all other reviewers who assisted us during 2012.

